# Efficacy and safety of pembrolizumab monotherapy in EGFR-mutant squamous cell lung cancer with PD-L1 over-expression: A case report

**DOI:** 10.1097/MD.0000000000030099

**Published:** 2022-08-19

**Authors:** Qiu-Xia Liu, Jian-Guo Wei, Yi-Yi Chen, Jian-Fang Wang

**Affiliations:** a Department of Medical Oncology; b Department of Pathology, Shaoxing People’s Hospital, Shaoxing 312000, Zhejiang Province; c Wenzhou Medical University, Department of Clinical Medicine, Wenzhou 325000, Zhejiang Province, China.

**Keywords:** EGFR mutation, Immunotherapy, PD-L1 over-expression, primary resistance, squamous cell lung cancer

## Abstract

**Background:**

Epidermal growth factor receptor (EGFR)-mutant nonsmall cell lung cancer (NSCLC) patients are less likely to be programmed death-ligand 1 (PD-L1)-positive compared with wild-type EGFR mutant tumors. Given the rarity of actionable driver genes in squamous cell lung cancer (SQCC), the frequency of SQCC patients simultaneously carrying EGFR driver gene mutation and having PD-L1 over-expression is extremely low. Studies on the effectiveness and safety of EGFR-TKIs or immune-checkpoint inhibitors (ICIs) in this subset of patients are lacking.

**Patient concerns:**

The patient suffered from coughing and chest pain for 1 month. A chest CT revealed a mass with a cavity in the right lung, enlarged mediastinal lymph nodes, diffuse pleural thickening in the right pleura, and pleural effusion of the right chest.

**Diagnosis:**

A pleural biopsy was performed using a video-assisted thoracoscope. The pathological examination revealed a poorly differentiated squamous cell carcinoma of lung. Further genetic testing identified exon 19 deletion mutation in EGFR with abundance of 0.27%. Meanwhile, immunohistochemical PD-L1 analysis showed a TPS of 90%.

**Interventions:**

The patient was initially resistant to EGFR-TKIs but exhibited a rapid and marked response to pembrolizumab.

**Outcomes:**

After 5 cycles of pembrolizumab monotherapy, the patient developed Grade 3 immune-related dermatitis, and ICI therapy was suspended.

**Conclusions:**

ICI monotherapy could be an effective therapy in SQCC patients with low-abundance of EGFR mutations and PD-L1 over-expression. However, close attention should be paid to immune-related adverse events.

## 1. Introduction

Epidermal growth factor receptor tyrosine-kinase inhibitors (EGFR-TKIs) have been used as the standard first-line treatment for advanced nonsmall-cell lung cancer (NSCLC) with an EGFR sensitive mutation (L858R or exon 19 deletion).^[1]^ In addition, there is overwhelming evidence demonstrating that a remarkable and durable response to the immune-checkpoint inhibitors (ICIs) targeting the programmed cell death-1 (PD-1) receptor and programmed death-ligand 1 (PD-L1) pathways has been achieved in NSCLC patients, especially those with high PD-L1 expression.^[2–5]^ However, the majority of clinical trials examining the efficacy of ICIs in NSCLC have excluded patients harboring oncogenic driver mutations.^[2–4]^ Moreover, data from some reports have suggested that ICI monotherapy is much less effective in NSCLC patients with EGFR driver alterations than those with no driver gene mutations, whether as first-line or subsequent-line therapy.^[5–9]^ Thus, due to the encouraging effects of EGFR-TKIs but the poor efficacy of single-agent ICI in NSCLC patients harboring the EGFR sensitive mutation, ICI monotherapy is generally not recommended in this subset of patients. However, as only a small percentage of squamous cell lung cancer (SQCC) patients carry oncogenic drivers, almost all the data on this subject are from studies on lung adenocarcinoma, and there is insufficient evidence on the efficacy of ICIs in SQCC with driver mutations.

We report a patient with advanced SQCC with an EGFR exon 19 deletion mutation and a PD-L1 Tumor Proportion Score (TPS) of 90%. He was initially resistant to EGFR-TKI treatment but showed a marked response to pembrolizumab.

Ethics approval for this study was obtained from the Shaoxing People’s Hospital Ethics Committee, and the patient provided informed consent for the publication of this case report and the accompanying images.

## 2. Case presentation

**Figure 1.**
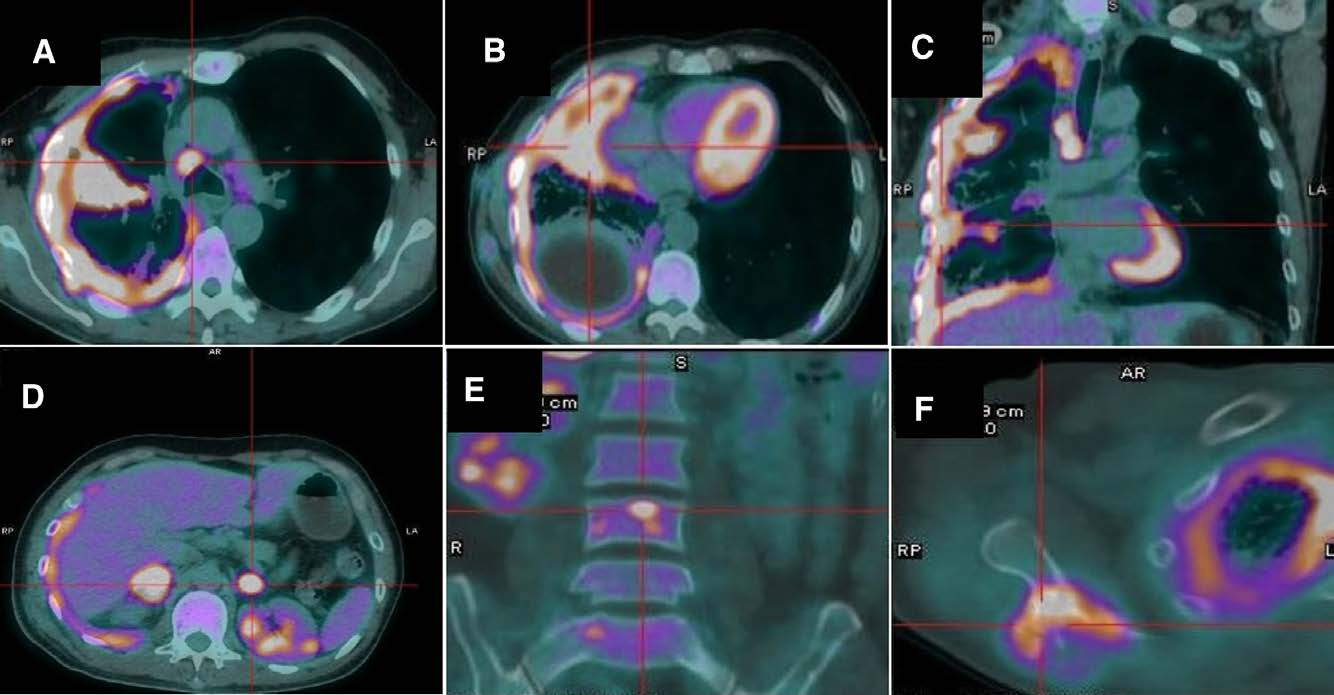
PET-CT scanning. PET-CT showing an abnormal increase in glucose metabolism of the right lung mass, enlarged mediastinal lymph nodes, and diffuse thickening of right pleura. In addition, systemic metastases in bilateral adrenal glands, right scapula, and multiple vertebrae were revealed.

**Figure 2.**
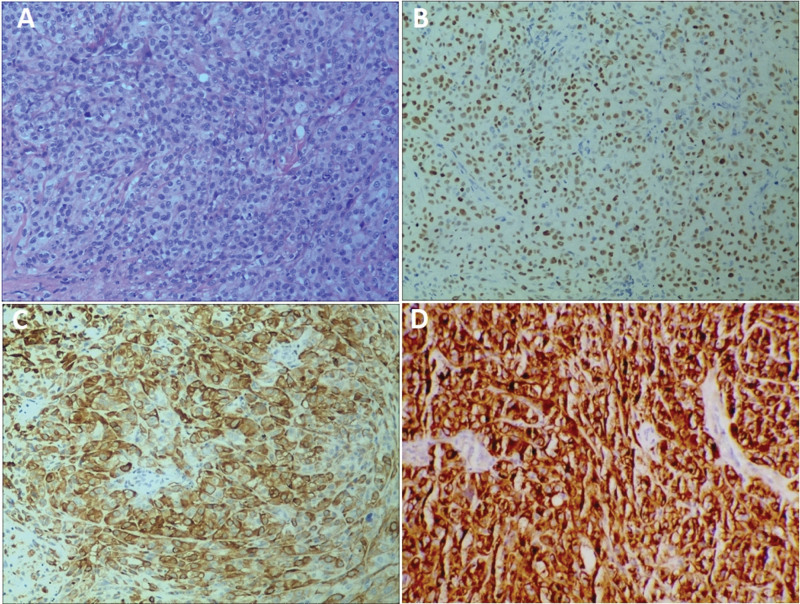
Immunohistochemistry findings. A: H&E staining showing tumor cells present in a diffuse and uniform patchy distribution, with abundant cytoplasm, and arranged into nests of different sizes (100×). B: Immunohistochemistry revealing diffuse expression of P40 (100×). C: Immunohistochemistry revealing diffuse expression of CK5/6 (400×). D: PD-L1 protein expression determined by the Tumor Proportion Score (TPS) was 90%.

A 54-year-old man with a 30 pack-year smoking history, who had been suffering from coughing and chest pain for 1 month, was admitted to our hospital in November 2019. A chest CT revealed a 2 cm mass with a cavity in the middle lobe of the right lung, enlarged mediastinal lymph nodes, diffuse pleural thickening in the right pleura, and pleural effusion of the right chest. A subsequent positron emission tomography-computed tomography (PET-CT) scan confirmed an abnormal increase of glucose metabolism in the right lung mass and thick pleura (Fig. 1A–C). It also revealed systemic metastases in the bilateral adrenal glands, right scapula, and multiple vertebrae (Fig. 1D–F). Laboratory findings were within the normal ranges, except for a high carbohydrate antigen 125 level of 490.2 U/ml. Thoracocentesis was performed, but no tumor cells were discovered in the drained pleural fluid after repeated exfoliative cytology examinations. Next, a bronchoscopy examination showed compressed bronchiostenosis at the opening of the right middle bronchus, but pathological results revealed no malignant cells. Then, a CT-guided fine-needle biopsy of the mass was conducted, and the pathology of the biopsy specimen only showed coagulative necrosis and abnormal lung tissue. Finally, a pleural biopsy was performed using a video-assisted thoracoscope and the biopsy specimen presented malignant tumor cells. Immunohistochemical staining showed that the tumor was negative for TTF-1 and Napsin A, but positive for P40 and CK5/6, indicating a pathological diagnosis of poorly differentiated squamous cell carcinoma (Fig. 2A–C). Accordingly, the patient was diagnosed with squamous cell lung cancer (T3N2M1c, stage IVB by the American Joint Committee on Cancer, 8th edition). EGFR exon 19 deletion mutations were detected by the ARMS-PCR procedure. The assessment of PD-L1 expression using antibody 22C3 (Dako pharmDx) showed a TPS of 90% (Fig. 2D).

**Figure 3.**
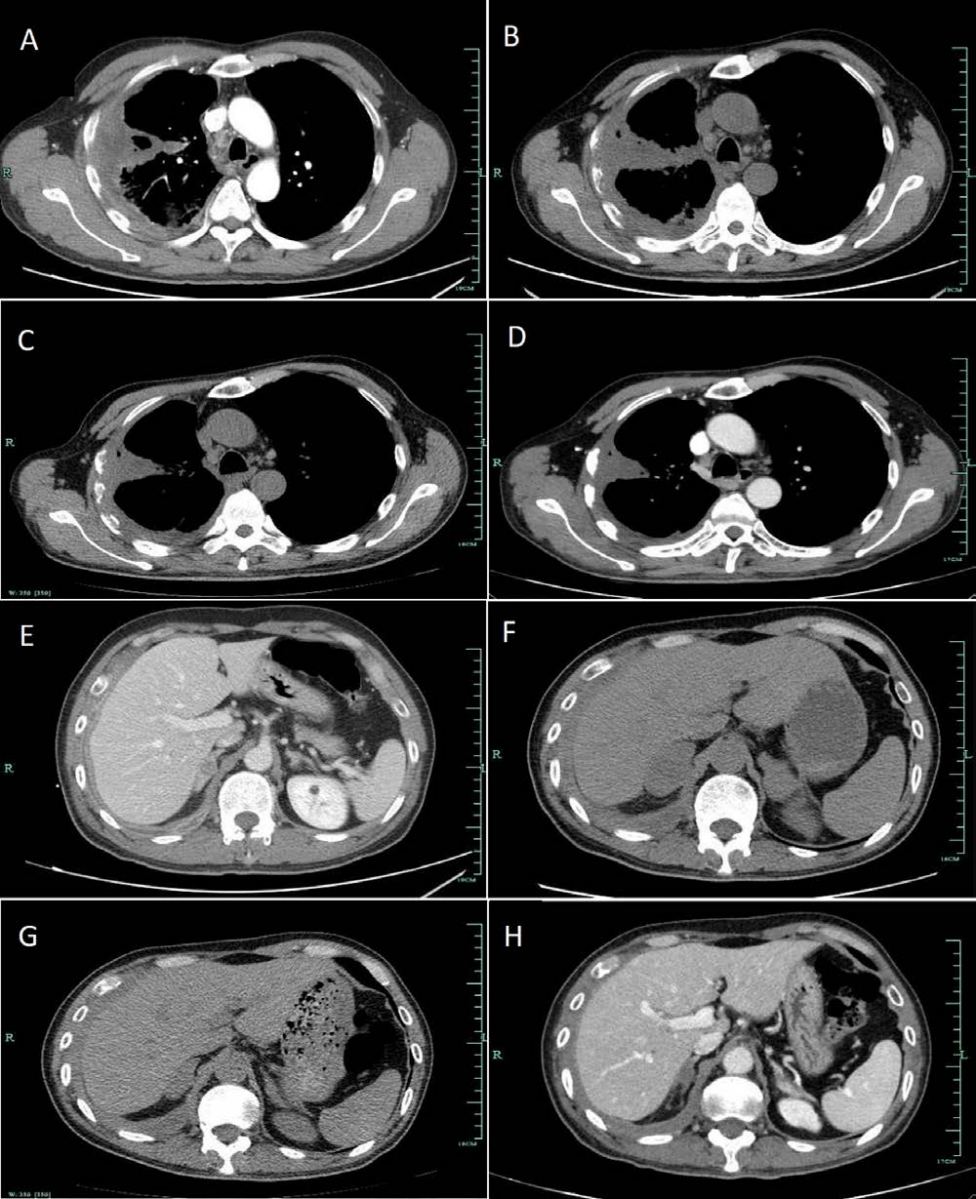
Changes in lesions of the lung (A-D) and bilateral adrenal glands (E-H) on CT during treatment with EGFR-TKIs and pembrolizumab monotherapy. A/E: pretreatment. B/F: EGFR-TKI targeted therapy for 2 months. C/G: Pembrolizumab monotherapy for 1 cycle. D/H: Pembrolizumab monotherapy for 5 cycles.

**Figure 4.**
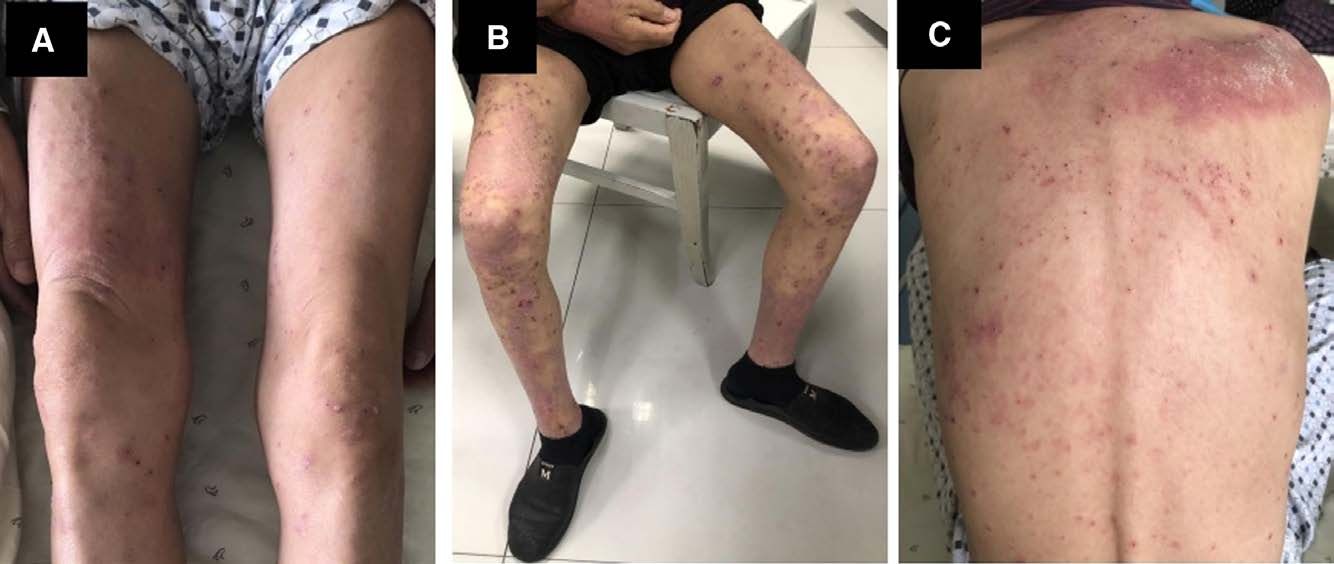
The status of body rash after pembrolizumab monotherapy. A: Grade 1 maculopapular rash appeared on the patient’s legs after 5 cycles of ICI monotherapy. B/C: The rash aggravated to Grade 3 and spread to nearly all of his legs and back a week later.

**Figure 5.**
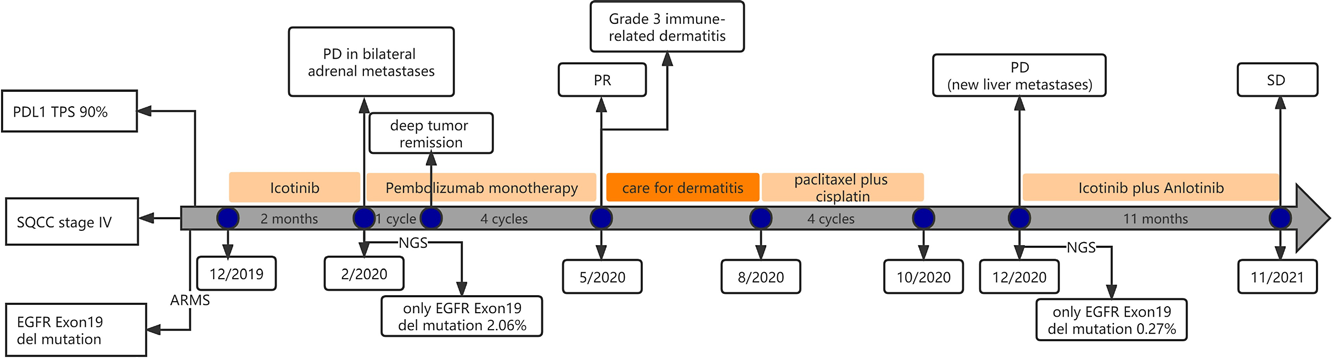
Timeline showing the history of treatment in this patient.

Icotinib was administered as the first-line targeted therapy on November 12, 2019. In addition, the patient underwent palliative radiotherapy to the right scapula. Two months later, chest CT showed that there was no obvious change in the lung mass, pleural thickening, and enlarged lymph nodes, but there was significant progression in the bilateral adrenal metastases. A next-generation sequencing gene test of the plasma covering 9 genes was conducted to exclude co-current gene mutations. The results confirmed that the EGFR exon 19 deletion was the only genetic mutation, and the fractional abundance was 2.06%. After 2 months of targeted therapy, the patient experienced serious fatigue and lost 4 kg in weight. We then chose pembrolizumab monotherapy as the second-line therapy at a dose of 100 mg every 3 weeks. After 1 dose of pembrolizumab, deep tumor remission was achieved (Fig. 3). The patient subsequently received another 4 cycles of pembrolizumab. The lesions in the chest and adrenal glands significantly decreased, resulting in partial remission (PR) according to the Response Evaluation Criteria in Solid Tumors Version 1.1 standard (Fig. 3). However, after 5 cycles of immune-checkpoint therapy, a rash characterized by the presence of macules and papules appeared on his legs, covering <10% of the body surface area (Fig. 4A). This was determined to be Grade 1 according to the National Comprehensive Cancer Network guidelines for immune-checkpoint inhibitor-related toxicities, and topical steroids were applied to the affected areas. However, his skin condition continued to worsen, and a week later the maculopapular rash became more intense and spread to nearly all of his legs, back and parts of his chest (Fig. 4B,C). It now covered more than 30% of his body and was therefore classified as Grade 3. The rash was treated with prednisone at the dose of 1 mg/kg/d. Initially, the rash reduced slightly, but the medication quickly became ineffective even when the dose was doubled. The patient then attended another hospital for traditional Chinese medicine treatment for the rash, and the ICI therapy was suspended. When the rash was controlled, he received 4 cycles of chemotherapy with paclitaxel (albumin-bound) plus cisplatin. He underwent another gene test of plasma after liver metastases appeared in December 2020, and the results still showed that there were no other oncogenic alternations except the EGFR exon 19 deletion mutation with abundance of 0.27%. Then, icotinib plus anlotinib (an oral small-molecule tyrosine kinase inhibitor of VEGFR, PDGFR, FGFR, c-Kit, and Met) were prescribed. To date, he has taken the combined targeted drugs for eleven months. The timeline for the treatment of this patient is shown in Figure 5.

He was in good condition at the last follow-up in November 2021, and all of the lesions were stable compared with his condition in December 2020.

## 3. Discussion

SQCC is a distinct histologic subtype of NSCLC and the EGFR mutation rate in this subset of patients is <4%.^[10]^ A pooled analysis of 3969 patients from 18 studies indicated that EGFR-mutant NSCLC patients are less likely to be PD-L1-positive compared with wild-type EGFR mutant tumors with an odds ratio of 0.59 (*P* < .02).^[11]^ Deepa *et al* also reported that PD-L1 ≥ 50% seldom overlaps with the presence of driver oncogene EGFR.^[12]^ Given the rarity of actionable driver genes in SQCC, the frequency of SQCC patients simultaneously carrying the EGFR driver gene mutation and having PD-L1 over-expression is extremely low. To our knowledge, only 1 case report related to this subject has been published so far.^[13]^

Multiple clinical studies have already demonstrated that SQCC patients are generally less sensitive to EGFR-TKI therapy than those with adenocarcinoma. It has been reported that the progression-free survival (PFS) for EGFR-TKI treatment in EGFR mutant SQCC is 3 months, and results from a pooled analysis indicated an objective response rate (ORR) and disease control rate of 30% and 70%, respectively.^[14]^ Nonetheless, previous studies have revealed that the abundance of the EGFR mutation may be related to the efficacy of EGFR-TKIs. NSCLC patients with low-abundance EGFR mutation may experience reduced ORR and shorter PFS time compared to groups with high-abundance EGFR mutation.^[15,16]^ Data from these studies indicate that treatment with EGFR-TKI for the patient in our case would probably be relatively ineffective, and the patient did indeed experience primary resistance to EGFR-TKI therapy.

It has been reported that approximately 10–30% of EGFR-mutant advanced NSCLC patients do not exhibit an objective response to EGFR-TKIs. Many factors contribute to the primary resistance of NSCLC with the sensitive EGFR mutation to EGFR-TKIs. Firstly, co-occurring genetic alterations in T790M/TP53/RB1/PTEN/PIK3CA may play a role in primary resistance mechanisms to EGFR-TKIs.^[17,18]^ In this case, to exclude co-occurring genetic mutations, we conducted a next-generation sequencing-based gene panel test, but the result was negative. Alternatively, PD-L1 expression could be a progression factor for EGFR-TKI primary resistance. Several studies have retrospectively examined the relationship between PD-L1 expression and the efficacy of EGFR-TKIs, and most of them have revealed that PD-L1 expression has a negative impact on the clinical outcome of EGFR mutant patients.^[19–22]^

Indeed, for NSCLC patients with the EGFR mutation, not only did high PD-L1 expression negatively impact the efficacy of EGFR-TKIs, but PD-1 inhibitor therapy was also reported to be less effective in this subgroup of patients compared to those with wild-type EGFR as first-line therapy. A clinical Phase II trial of pembrolizumab in TKI-naïve EGFR-mutant NSCLC patients with positive PD-L1 showed no response.^[9]^ In addition, a pooled analysis of CheckMate 057, KEYNOTE-010, POPULAR, and OAK clinical trials revealed that ICI monotherapy provided no benefits over docetaxel as second-line treatment for EGFR-mutant NSCLC patients with failed EGFR-TKI therapy.^[7]^ Moreover, data from the ImmunoTarget trial showed that the ORR and PFS of EGFR-mutant NSCLC patients treated with ICI monotherapy at any line of treatment were 12% and 2.1 m, respectively.^[8]^

Although the efficacy of ICI in NSCLC patients harboring oncogenic alterations is poor, the PD-L1 status is still believed to be the most effective predictive biomarker for identifying patients most likely to benefit from ICI.^[8,23]^ Except high PD-L1 levels, Limited data indicated that smoking history,^[24]^ L858R mutation subtype,^[25]^ or uncommon EGFR mutation type^[26,27]^ are also associated with better outcomes following treatment with immunotherapy. A recent study retrospectively analyzed advanced NSCLC patients with resistance to EGFR-TKIs who received ICI treatment. The results indicated that patients with a short PFS to EGFR-TKIs showed a better response to subsequent anti-PD-1/PD-L1-based immunotherapy in EGFR-mutation NSCLC.^[28]^ Considering these viewpoints, our patient could benefit from ICI due to his high PD-L1 expression, history of smoking, and short PFS to EGFR-TKIs.

In terms of immune-related toxicity, several studies state that PD-L1 inhibitor and EGFR-TKI combination therapy may be associated with an increased risk of toxicity.^[29,30]^ Conversely, another study has found that erlotinib plus atezolizumab or erlotinib plus nivolumab did not demonstrate excess toxicity.^[31]^ With regard to PD-L1 inhibitor and EGFR-TKI sequential therapy, it has been reported that treatment with sequential PD-L1 blockade followed by osimertinib resulted in a severe immune-related adverse event.^[32]^ Despite this, no severe immune-related adverse events were identified among patients treated with osimertinib followed by PD-L1 or PD-L1 followed by afatinib or erlotinib.^[32]^ However, our patient developed Grade 3 immune-associated dermatitis when receiving pembrolizumab after failed EGFR-TKI therapy. It was difficult to distinguish whether the dermatitis was only related to the immunotherapy or was also associated with the increased risk of adverse events due to the PD-1 inhibitor treatment after EGFR-TKI therapy.

This report indicates that high PD-L1 expression may correlate with primary resistance to EGFR-TKIs in treating naïve advanced NSCLC with EGFR mutation. For SQCC patients, if they have lower EGFR mutation fractions, but higher PD-L1 expression, ICI monotherapy may be a therapeutic option. During the ICIs treatment, close attention should be paid to immune-related adverse events. However, further investigations into the optimal treatment for SQCC with the EGFR mutation and PD-L1 over-expression are required to confirm this hypothesis.

## Acknowledgments

The authors thank the patient and her family for signing informed consent for publication.

## Author contributions

Qiu-Xia Liu conceptualized the study and drafted the manuscript; Jian-Guo Wei and Yi-Yi Chen collected and analyzed the patient data; Jian-Fang Wang supervised the manuscript preparation. All authors have read and approved the final version to be submitted.
